# AGE-FRIENDLY PROGRESS WITH THE HEALTHYAGINGDATAREPORTS.ORG: NOTES FROM MA, RI, NH, CT, ME, MS, AND WY

**DOI:** 10.1093/geroni/igae098.0300

**Published:** 2024-12-31

**Authors:** Elizabeth Dugan

**Affiliations:** Chair

## Abstract

This symposium celebrates ten years of collaboration to translate data into high impact tools for stakeholders forming coalitions, identifying priorities, and working to make their state or community age-friendly. The session will review the tools included in the healthyagingdatareports.org website and highlight the exciting and replicable age-friendly work underway in Massachusetts, New Hampshire, Mississippi, Maine, Connecticut, Rhode Island, and Wyoming. We have found that identifying health disparities by gender, race, or geography may be a key step in addressing health equity. The process involved in each state and how the reports were launched to generate awareness and enthusiasm for each state’s age-friendly priorities are described. Dugan (chair) will present an overview of the data reports (community-engagement, data sources, indicator calculation methods, hierarchical approach to reporting, data visualization tools, dissemination efforts, and on-going technical support). Then leaders from Massachusetts (Fuccione), New Hampshire (Rabalais), Mississippi (White), and Wyoming (Vincenti) will share an overview of their state’s efforts to create longevity-ready communities and to promote healthy aging. They will also describe how their coalitions or collaboratives were formed and supported, how to identify the key stakeholders and various outreach approaches. State leaders will describe how the data report tools support their advocacy and/or outreach. Reflections on how age-friendly efforts are sustained and grow will be discussed. Attendees and presenters will then have a question and answer time to discuss how others could join the movement.

